# Supplementary material for: V-Porphyrins Encapsulated or Supported on Siliceous Materials: Synthesis, Characterization, and Photoelectrochemical Properties
Source: Materials (Basel). 2022 Oct 25;15(21):7473. doi: 10.3390/ma15217473 (PMC9658604; doi:10.3390/ma15217473)

Supplementary Materials

V-porphyrins encapsulated or supported on siliceous materials: synthesis characterization and photoelectrochemical properties

Zhannur K. Myltykbayeva[1,2], Anar Seysembekova[1,2], Beatriz M. Moreno[1], Rita Sánchez-Tovar[3], Alejandro Vidal-Moya[1], Benjamín Solsona[3,*], José M. López Nieto[1,*]

1 Instituto de Tecnología Química, Universitat Politècnica de València-Consejo Superior de Investigaciones Científicas, Avenida de los Naranjos s/n, 46022 Valencia, Spain

2 Al-Farabi Kazakh National University, 71 Al-Farabi Ave., Almaty 050040, Kazakhstan

3 Departament d'Enginyeria Química, Universitat de València, Av. de les Universitats, s/n, 46100 Burjassot, Spain

* Correspondence: benjamin.solsona@uv.es (B.S.); jmlopez@itq.upv.es (J.M.L.N.)

Figure S1. Scheme of the synthesis of mesoporous SBA-15.

Figure S2. **20VP@SBA** sample: a) after filtered and washed; b) after dried at 60 ºC overnight.

Figure S3. Structure of both a mesoporous SBA-15 materials and VTPP, as well as VTPP encapsulated on mesoporous material.

Figure S4. XRD patterns of catalysts: a) Pure VTPP; b) SiO_2-supported VTPP catalysts (with a 20wt% of VTPP).

Figure S5. Nitrogen adsorption isotherms (A) and desorption (B) of: a) support (mesoporous materials, after calcination at 500ºC); b) 20VTPP@SBA catalyst. Characteristics in Table 1.

Figure S6. A) FTIR spectra of support and catalysts: a) SBA-15; b) 20VTPP@SBA; c) 20VTPP/SiO_2; d) Pure VTPP. B) FTIR spectra in the 3750-2750 cm^{-1} region. C) FTIR spectra in the 200-500 cm^{-1} region.

Figure S7. Diffuse Reflectance (UV-vis) spectra of pure **VTPP** (as received) and **VTPP** diluted in CH_2Cl_2 (3 mg VPTT/30g CH_2Cl_2).

Figure S8. Diffuse Reflectance (UV-vis) spectra of SiO_2-supported VTPP catalysts, with VTPP contents of: a) 20 wt.% of VTPP; b) 10 wt.% of VTPP; c) 5 wt.% of VTPP.

Figure S9. EPR spectra of pure **VTPP** and **20VP@SBA** catalysts.

Figure S1. Scheme of the synthesis of mesoporous SBA-15.

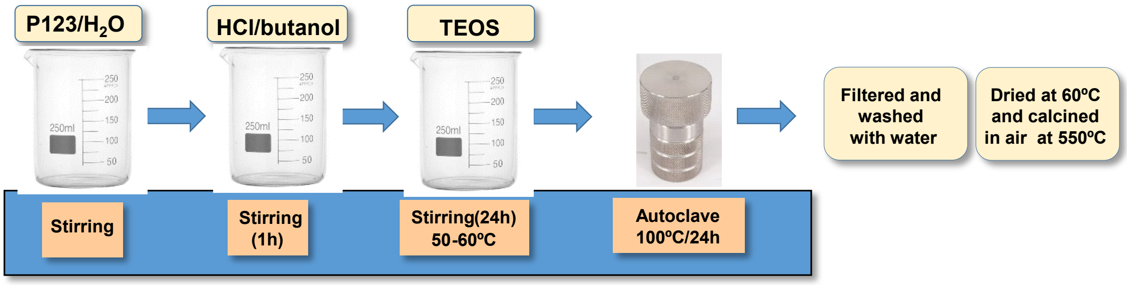

Figure S2. **20VP@SBA** sample: a) after filtered and washed; b) after dried at 60 ºC overnight.

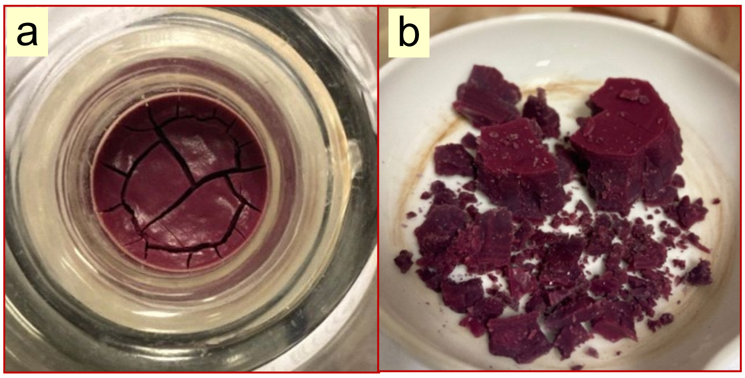

Figure S3. Structure of both a mesoporous SBA-15 materials and VTPP, as well as VTPP encapsulated on mesoporous material.

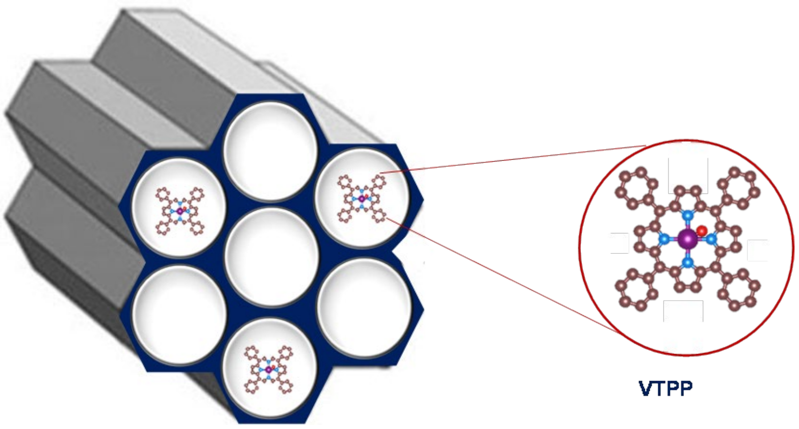

Figure S4. XRD patterns of catalysts: a) Pure VTPP; b) SiO_2-supported VTPP catalysts (with a 20wt% of VTPP).

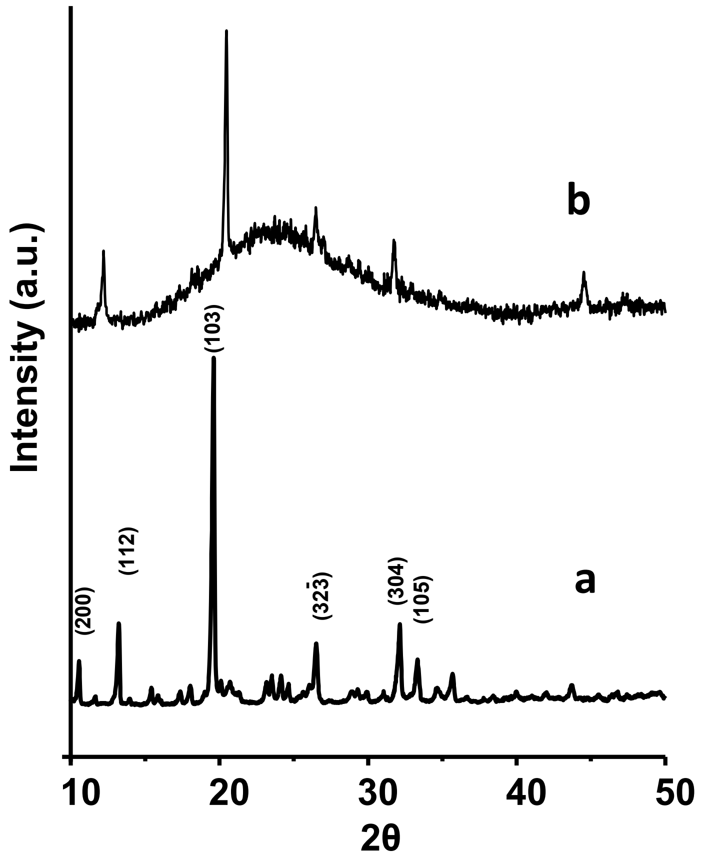

Figure S5. Nitrogen adsorption isotherms and desorption of: a) SBA-15 support (mesoporous materials, after calcination at 500ºC); b) Nb-containing SBA-15 support (mesoporous materials, after calcination at 500ºC).

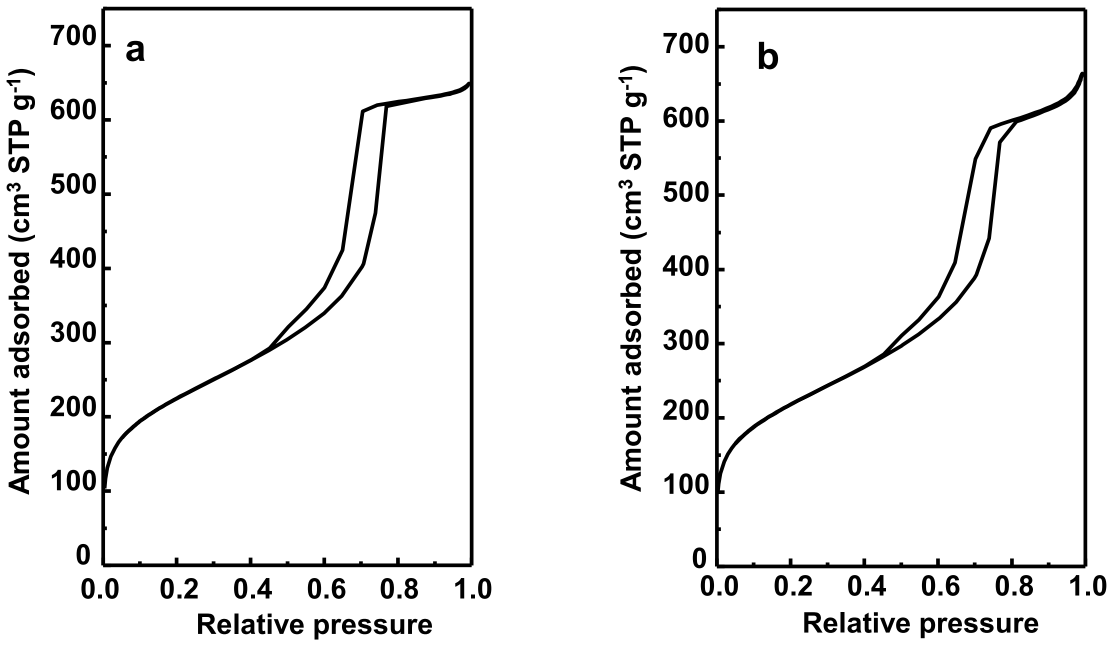

Figure S6. A) FTIR spectra of support and catalysts: a) SBA-15; b) 20VTPP@SBA; c) 20VTPP/SiO_2; d) Pure VTPP. B) FTIR spectra in the 3750-2750 cm^{-1} region. C) FTIR spectra in the 200-500 cm^{-1} region.

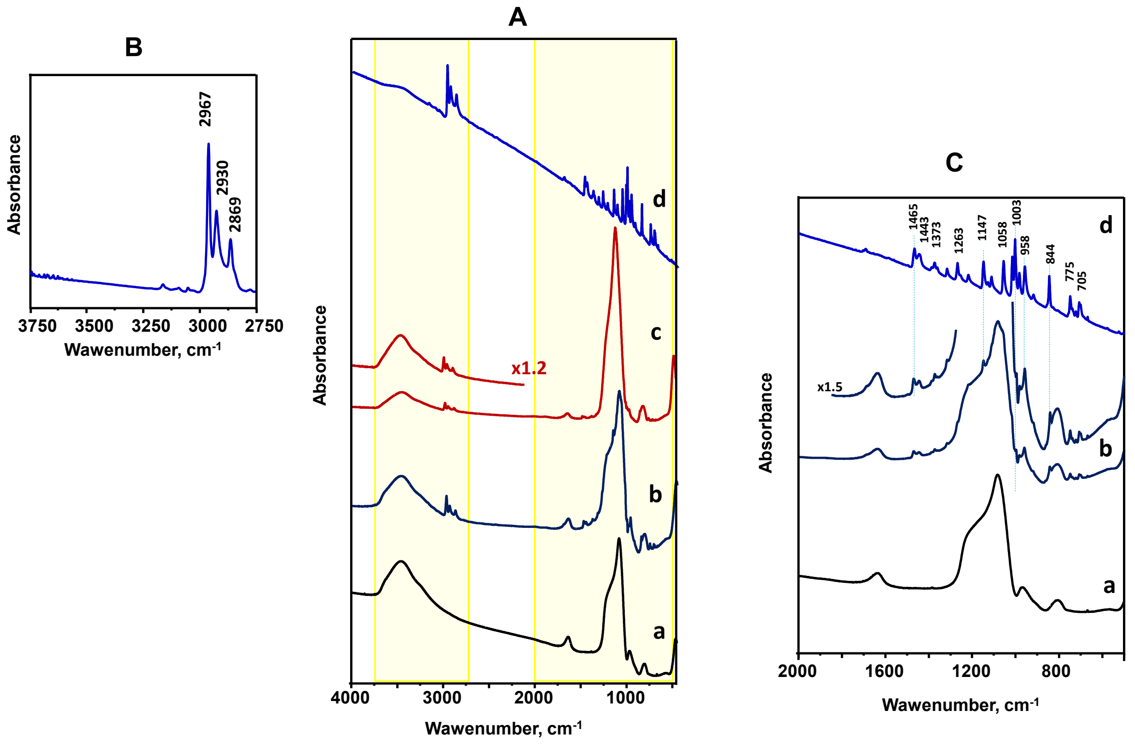

Figure S7. Diffuse Reflectance (UV-vis) spectra of pure **VTPP** (as received) and **VTPP** diluted in CH_2Cl_2 (3 mg VPTT/30g CH_2Cl_2).

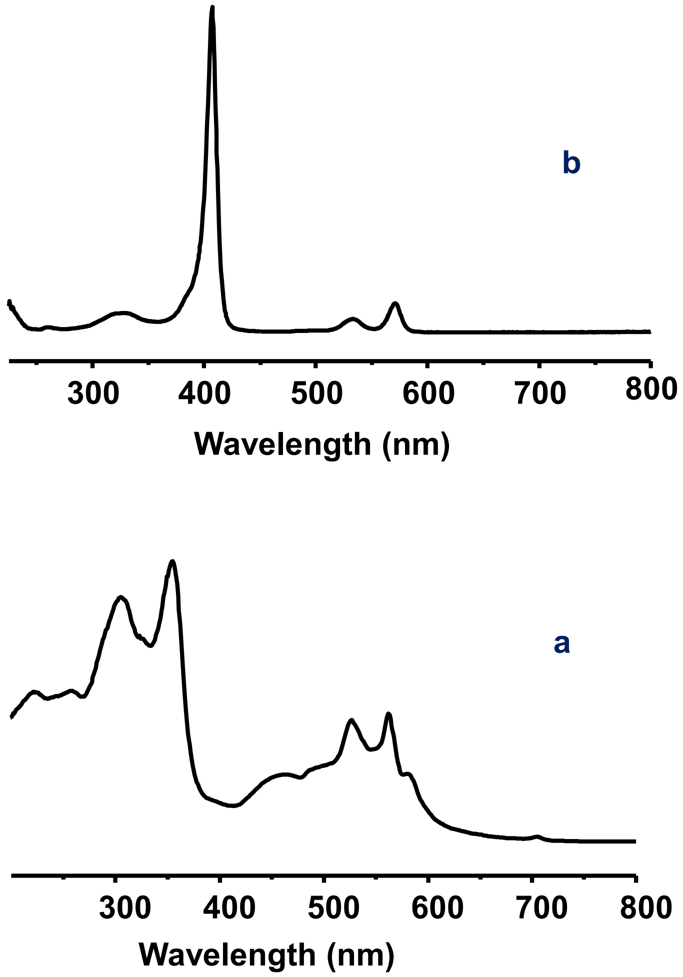

Figure S8. Diffuse Reflectance (UV-vis) spectra of SiO_2-supported VTPP catalysts, with VTPP contents of: a) 20 wt.% of VTPP; b) 10 wt.% of VTPP; c) 5 wt.% of VTPP.

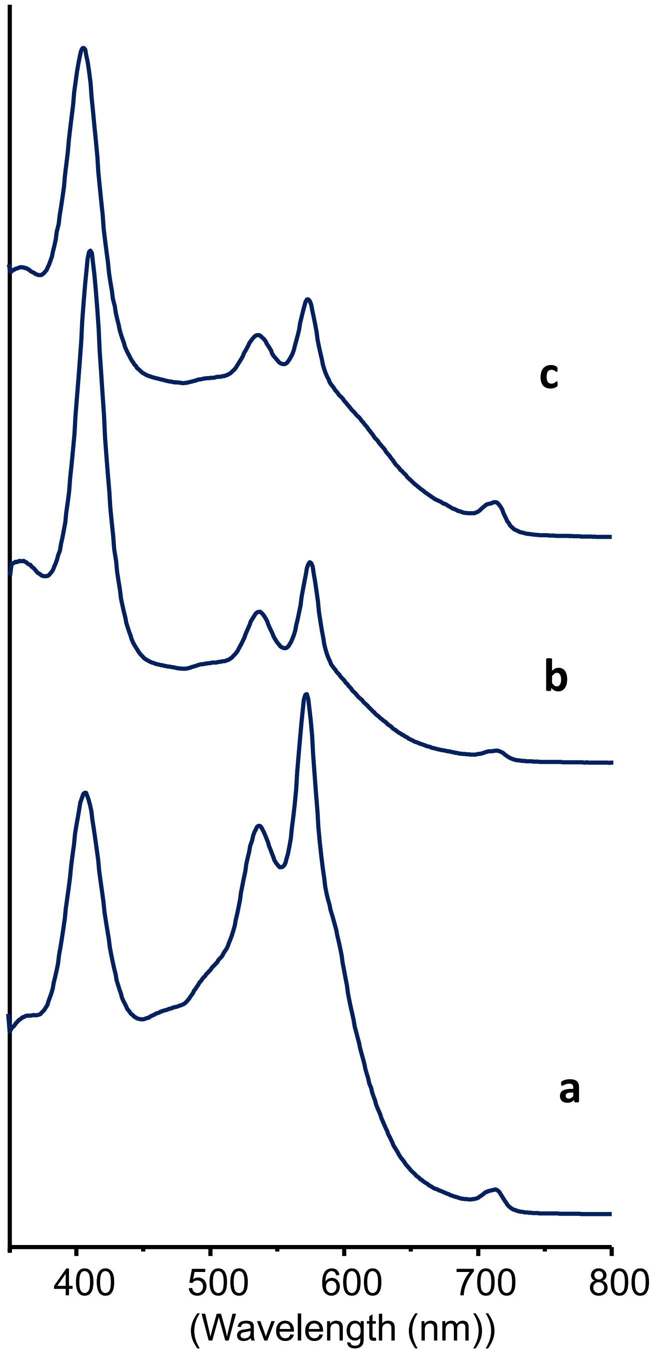

Figure S9. EPR spectra of pure **VTPP** and **20VP@SBA** catalysts.

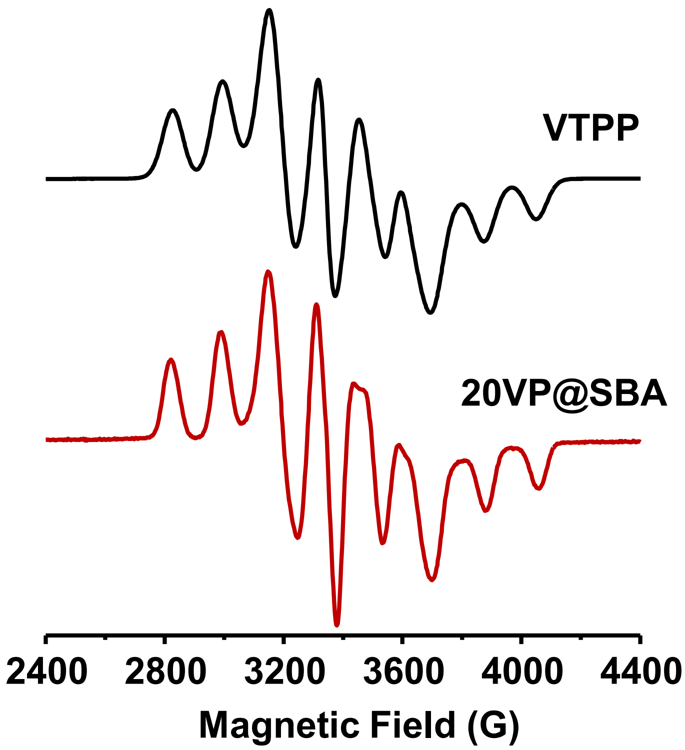

Supplement: Supplementary file 1 [file materials-15-07473-s001.zip › materials-1963360-supplementary.pdf]
